# Robotic *versus* laparoscopic hepatectomy: meta-analysis of propensity-score matched studies

**DOI:** 10.1093/bjsopen/zrae141

**Published:** 2025-04-01

**Authors:** Piao Wang, Dan Zhang, Bin Huang, Wen-Hao Zhou, Chang-Song Wang, Shao-Yong Zhao, Song Su, Xiao-Zhong Jiang

**Affiliations:** Department of Hepatobiliary and Pancreatic Surgery, The Second People’s Hospital of Yibin, Yibin, Sichuan, China; Department of Thyroid and Breast Surgery, The Third People’s Hospital of Yibin, Yibin, Sichuan, China; Department of Hepatobiliary and Pancreatic Surgery, The Second People’s Hospital of Yibin, Yibin, Sichuan, China; Department of Hepatobiliary and Pancreatic Surgery, The Second People’s Hospital of Yibin, Yibin, Sichuan, China; Department of Hepatobiliary and Pancreatic Surgery, The Second People’s Hospital of Yibin, Yibin, Sichuan, China; Department of Hepatobiliary and Pancreatic Surgery, The Second People’s Hospital of Yibin, Yibin, Sichuan, China; Department of General Surgery (Hepatobiliary Surgery), The Affiliated Hospital of Southwest Medical University, Luzhou, Sichuan, China; Department of Hepatobiliary and Pancreatic Surgery, The Second People’s Hospital of Yibin, Yibin, Sichuan, China

## Abstract

**Background:**

Robotic techniques can theoretically overcome the limitations of laparoscopic liver resection and are currently recognized as safe options; however, it is not known which approach is better. The purpose of this study was to compare the advantages of robotic hepatectomy and laparoscopic hepatectomy.

**Methods:**

Electronic databases (the Cochrane Library, PubMed (MEDLINE), Embase and Web of Science) were systematically searched from January 2000 to August 2023 for eligible studies that compared robotic hepatectomy and laparoscopic hepatectomy. Studies that met the inclusion criteria were then reviewed systematically. The reported data were aggregated statistically using RevMan 5.4 software. The parameters of interest included intraoperative, postoperative, survival and financial outcomes. Subgroup analysis was performed according to the type and difficulty level of hepatectomy and the study setting.

**Results:**

A total of 26 propensity-score matching comparative trials met the inclusion criteria, which comprised 9355 participants (robotic hepatectomy *versus* laparoscopic hepatectomy: 3938 *versus* 5417) in the meta-analysis. For surgical outcomes, lower blood loss, lower open conversion rate and higher R0 resection rate were observed in the robotic hepatectomy group compared with the laparoscopic hepatectomy group (mean difference (MD) −86.22, 95% c.i. −116.49 to −55.95, *I*² = 87%, *P* < 0.001; OR 0.51, 95% c.i. 0.38 to 0.69, *I*² = 40%, *P* < 0.001; OR 1.31, 95% c.i. 1.03 to 1.67, *I*² = 0%, *P* = 0.030 respectively). The lower blood loss (major hepatectomy group: MD −56.88, 95% c.i. −109.09 to −4.28, *I*² = 76%, *P* = 0.030; IWATE score (advanced/expert more than 80%) group: MD −0.61, 95% c.i. −1.14 to −0.08, *I*² = 95%, *P* < 0.001) and lower open conversion rate (major hepatectomy group: OR 0.41, 95% c.i. 0.30 to 0.56, *I*² = 0%, *P* < 0.001; IWATE score (advanced/expert less than 80%) group: OR 0.52, 95% c.i. 0.36 to 0.75, *I*² = 0%, *P* = 0.659) advantage persisted across subgroup analyses.

**Conclusion:**

The robotic approach had advantages to laparoscopic in terms of lower blood loss and reduced rates of open conversion, especially in difficult hepatectomies.

## Teaser

The advantages of robotic hepatectomy for laparoscopic hepatectomy remain unclear and require clarification. This is the largest meta-analysis on this topic, with the largest number of patients; it is also the first meta-analysis to compare robotic hepatectomy with laparoscopic hepatectomy in patients with different difficulty levels of hepatectomy to date. The robotic approach has significant advantages over laparoscopic in surgical outcomes, especially in terms of lower blood loss and conversion to open.

## Introduction

To date, both randomized clinical trials (RCTs) and international consensus have recommended the use of laparoscopic hepatectomy (LH) as the standard approach for the resection of benign and malignant liver tumours^1–3^. The LH approach is associated with reduced blood loss, faster rehabilitation and comparable oncological outcomes compared with the open approach^1–4^. The use of LH in complex liver resections is hindered by its inherent drawbacks, including the limited degrees of freedom (only four degrees), a prolonged learning curve, hand tremor and surgeon fatigue^5^. Robotic hepatectomy (RH) allows better dexterity and stability and is associated with a shorter learning curve, with several studies demonstrating its technical feasibility^6–8^. Although recent years have seen an exponential increase in the number of studies comparing the outcomes of RH and LH in different types of hepatectomy and difficulty levels^9–11^, high-level evidence is still missing. Despite the many theoretical advantages of robotic surgery, the advantages of RH relative to LH remain unclear and require clarification.

Several recent reviews and meta-analyses have compared the outcomes of robotic and laparoscopic liver resections^12–14^. Nevertheless, none of these has compared the long-term prognosis associated with these two surgical methods for liver malignancies. In addition, due to discrepancies in data and confounding variables in the included cohorts, it is difficult to draw reliable conclusions from these previous studies. In case-control studies, propensity-score matching (PSM) is often used to enhance comparability and reduce the effects of data inconsistencies and confounding variables, thus facilitating comparisons between the experimental and control groups^15^. There has been no meta-analysis that used PSM to compare the outcomes of RH and LH.

Thus, the purpose of this study was to conduct a meta-analysis using PSM to clarify the comparative short- and long-term benefits of RH and LH. The distribution of available studies in terms of hepatectomy type and IWATE score was assessed, and subgroup meta-analysis was performed by the grouping of like studies to increase the reliability of the evidence.

## Methods

This work was conducted in line with PRISMA (Preferred Reporting Items for Systematic Reviews and Meta-Analyses)^16^ and AMSTAR (Assessing the Methodological Quality of Systematic Reviews) guidelines^17^. This study was prospectively registered in the Prospective Register of Systematic Reviews (PROSPERO) (number: CRD42023464248).

### Literature search

A systematic search of the Cochrane Library, PubMed (MEDLINE), Embase and Web of Science databases was performed. All potentially relevant studies from January 2000 to August 2023 were identified. The following Medical Subject Headings (MeSH) terms were used: ‘hepatic’, ‘hepatectomy’, ‘hepatectomies’, ‘hepato’, ‘sectionectomy’, ‘robot’, ‘robotic’, ‘robotics’, ‘laparoscopic’, ‘laparoscopies’, ‘laparoscopy’. Additional studies were identified from hand-searching reference lists of all relevant articles.

### Inclusion and exclusion criteria

Articles included patients with a resectable liver disease (whether benign or malignant), hepatectomy performed robotically or laparoscopically (RH *versus* LH) and they were a prospective or retrospective study matched with PSM, published in the English language. The exclusion criteria were studies whose data could not be extracted, or animal experiments, reviews, guidelines, meta-analyses, case reports, abstracts, conference proceedings or expert opinions.

### Study selection and data extraction

The study selection and data extraction were conducted separately by two independent reviewers and the extracted data was cross-checked. In opposing views between the two reviewers, a third reviewer was invited to reconcile the differences. If the data were unclear, the corresponding author of the study was contacted by e-mail to obtain insight into the original data set.

Data extracted from each study were: study type, year of publication, area of publication, baseline characteristics and intervention measures of study subjects, critical factors for bias risk assessment and analysed outcomes such as operative time, blood loss, massive blood loss (>500 ml), intraoperative blood transfusion, open conversion rate, R0 resection, postoperative stay, postoperative morbidity rate, major morbidity rate (Clavien–Dindo grade greater than II) and overall survival (OS).

For subgroup analyses, the variables extracted were resection type and IWATE score. Resection type (minor or major) was classified using the Louisville Consensus meeting^18^. In subgroup analyses, if the proportion of patients who underwent minor hepatectomy in a study was more than 90%, this study was included in the minor group and vice versa for the major group. To better clarify the advantages of the two surgical approaches for different difficulty levels of hepatectomy, the patients were divided into two groups according to the IWATE scoring systems (advanced/expert more than 80% group or not, the proportion of advanced/expert level was reported by included studies).

### Literature quality assessment

Two investigators independently assessed the risk of bias in the included articles and cross-validated the results. The quality of non-randomized studies was evaluated using the Newcastle–Ottawa Scale (NOS). The standard included three categories—patient selection (4 points), comparability of the study groups (2 points), and ascertainment of exposure or outcome (3 points). Studies with a cumulative score greater than or equal to 7 points were considered to be of high quality.

### Statistical analysis

RevMan 5.4 software was used for the meta-analysis. The odds ratio (OR) was used to assess categorical variables and standardized mean difference (MD) was used for continuous variables. According to the method recommended by Guyot *et al.*^19^ based on the ‘Engauge Digitizer’ software, the survival rate data were re-extracted from Kaplan–Meier plots. The between-study heterogeneity was calculated using Higgins’ *I*^2^ statistic^20^. According to the heterogeneity among included studies, the random-effects or fixed-effects meta-analysis was conducted for outcomes. If significant interstudy heterogeneity was present (*I*² > 50%, *P* < 0.100), the source of heterogeneity was further analysed and subgroup analysis was used for processing. If heterogeneity could not be removed, the random-effects model (REM) was used for meta-analysis. All tests were two-tailed and a difference of *P* < 0.05 was considered statistically significant.

## Results

### Study selection and study characteristics

**Fig. 1:**
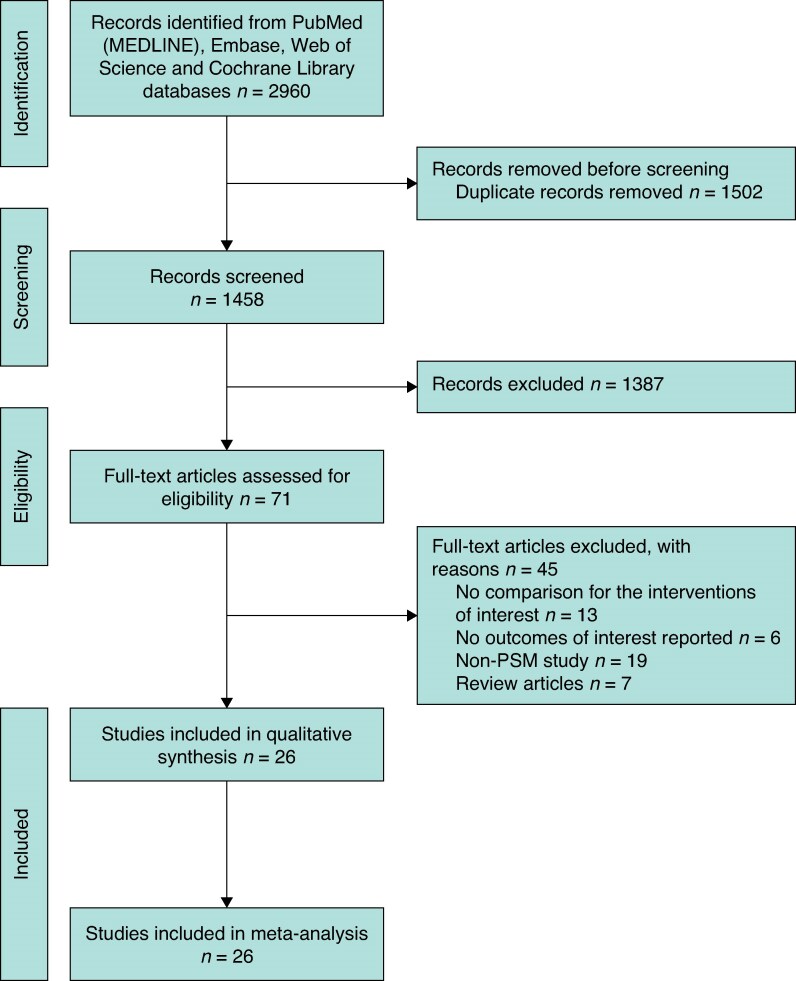
Steps of the process of screening documents

The designed strategy was conducted using literature selection, and 2960 related records were searched using search terms. After screening all titles and abstracts, a total of 71 full articles were retrieved, of which 45 were excluded because of missing inclusion criteria. In total, 26^7–11,21–41^ PSM studies, comprising 9355 participants (RH *versus* LH: 3938 *versus* 5417) were included in this meta-analysis. Steps of the screening document process are presented in the PRISMA flow chart (*Fig. 1*). Study characteristics are detailed in *Table 1*.

PSM, propensity-score matching.

**Table 1:** Summary characteristics of studies

Study author, year	Study design	Study area	Study interval	Number of patients	Age (years)*	Sex (male:female)*	Aetiology	Hepatectomy type
Montalti *et al.*, 2016^39^	Retrospective PSM	International	2008–2014	R:36 L:72	R:62(13) L:56.8(15)	R:(21:15) L:(39:33)	Mixed	Minor
Salloum *et al*., 2016^36^	Retrospective PSM	France	1997–2014	R:14 L:14	R:57(12) L:57(15)	–	Mixed	Minor
Lim *et al*., 2019^38^	Retrospective PSM	International	2011–2017	R:55 L:55	R:65(10) L:66(10)	R:(37:18) L:(41:14)	Malignancy	Mixed
Beard *et al*., 2020^37^	Retrospective PSM	International	2008–2016	R:115 L:115	R:61(11) L:61(12)	R:(76:39) L:(75:40)	Malignancy	Mixed
Chiow *et al.*, 2021^24^	Retrospective PSM	International	2010–2019	R:88 L:88	R:60(51–69) L:61(54–69)	R:(59:29) L:(64:24)	Mixed	Minor
Fagenson *et al*., 2021^28^	Retrospective PSM	USA	2014–2017	R:240 L:240	R:60(50–69) L:63(51–73)	R:(142:98) L:(140:100)	Mixed	Mixed
Aziz *et al*., 2022^22^	Retrospective PSM	USA	Before 2015	R:101 L:202	R:54.5(13.7) L:54.3(12.1)	R:(50:51) L:(101:101)	Malignancy	Mixed
Aziz *et al.*, 2022^22^**	Retrospective PSM	USA	2016–2018	R:331 L:662	R:54.5(13.7) L:54.3(12.1)	R:(168:163) L:(337:325)	Malignancy	Mixed
Chong *et al.*, 2022^8^	Retrospective PSM	International	2008–2020	R:220 L:220	R:61(52–69) L:63.00(55.00–71.00)	R:(139:81) L:(144:76)	Mixed	Major
Cipriani *et al*., 2022^26^†	Retrospective PSM	Italian	Before 2018	R:288 L:864	–	R:(168:120) L:(493:371)	Mixed	Mixed
D’Silva *et al.*, 2022^27^	Retrospective PSM	International	2010–2019	R:104 L:104	R:62(53–68) L:63(50–70)	R:(70:34) L:(68:36)	Mixed	Minor
Duong *et al.*, 2022^10^	Retrospective PSM	USA	2010–2015	R:123 L:369	R:62.0(55.0–70.0) L:62.0(57.0–69.0)	R:(88:35) L:(268:101)	Malignancy	Mixed
Kadam *et al*., 2022^29^	Retrospective PSM	International	2005–2020	R:296 L:296	R:61(52–67) L:61(51–70)	R:(191:105) L:(196:100)	Mixed	Mixed
Kamel *et al.*, 2022^30^	Retrospective PSM	USA	2010–2016	R:184 L:184	R:64(58–71) L:65(58–72)	R:(123:61) L:(125:59)	Malignancy	Mixed
Miller *et al*., 2022^33^	Retrospective PSM	USA	2014–2017	R:227 L:227	R:58.6(13.7) L:57.3(14.6)	R:(91:136) L:(76:151)	Mixed	Mixed
Rho *et al*., 2022^34^	Retrospective PSM	Korea	2016–2019	R:19 L:19	R:56.8(5.8) L:22.1(2.8)	R:(15:4) L:(14:5)	Benign	Mixed
Sucandy *et al*., 2022^35^	Retrospective PSM	International	2008–2020	R:164 L:164	R:62(17.3) L:63(15)	R:(100:64) L:(105:59)	Mixed	Major
Yang *et al.*, 2022^7^	Retrospective PSM	International	2010–2020	R:40 L:40	R:62(55–68) L:62(54–72)	R:(32:8) L:(33:7)	Mixed	Minor
Chen *et al*., 2023^9^	Retrospective PSM	China	2020–2022	R:41 L:41	R:53(13) L:54(12)	R:(24:17) L:(27:14)	Mixed	Minor
Cheung *et al.*, 2023^23^	Retrospective PSM	International	2002–2020	R:73 L:219	R:54(40–66) L:55(42–68)	R:(34:39) L:(105:114)	Mixed	Mixed
Chong *et al*., 2023^25^	Retrospective PSM	International	2006–2020	R:160 L:160	R:60(14) L:61(17)	R:(111:49) L:(115:45)	Mixed	Minor
Kato *et al*., 2023^31^	Retrospective PSM	Japan	2010–2022	R:31 L:31	R:72(21–82) L:70(36–83)	R:(25:6) L:(25:6)	Malignant	Mixed
Kwak *et al*., 2023^32^	Retrospective PSM	International	2003–2020	R:48 L:48	R:62(53–68) L:63(47–68)	R:(20:28) L:(20:28)	Benign	Mixed
Liu *et al*., 2023^11^	Retrospective PSM	International	2008–2021	R:841 L:841	–	–	Mixed	Major
Zhang *et al.*, 2023^41^	Retrospective PSM	China	2015–2021	R:43 L:86	R:48(26–62) L:49(27–66)	R:(13:30) L:(26:60)	Benign	Mixed
Zhu *et al*., 2023^40^	Prospective PSM	China	2015–2016	R:56 L:56	R:52(28–72) L:53(24–72)	R:(45:11) L:(47:9)	Malignant	Mixed

^*^Values are mean(s.d.) or mean (i.q.r.). **Different studies by the same author. †Three cohorts of varying difficulty were included within this study. PSM, propensity-score matched; L, laparoscopic; R, robotic.

### Quality assessment and publication bias

The quality evaluation of individual studies is shown in *Table S1* and all included studies were of high quality (NOS score equal to or greater than 7).

The funnel plots of this study created on open conversion rate and postoperative day are shown in *Fig. S1A, B*. The points are inside the limits of the 95% c.i. and are distributed more evenly about the vertical, which suggests there was no publication bias.

### The results of meta-analyses

#### Intraoperative outcomes

**Fig. 2:**
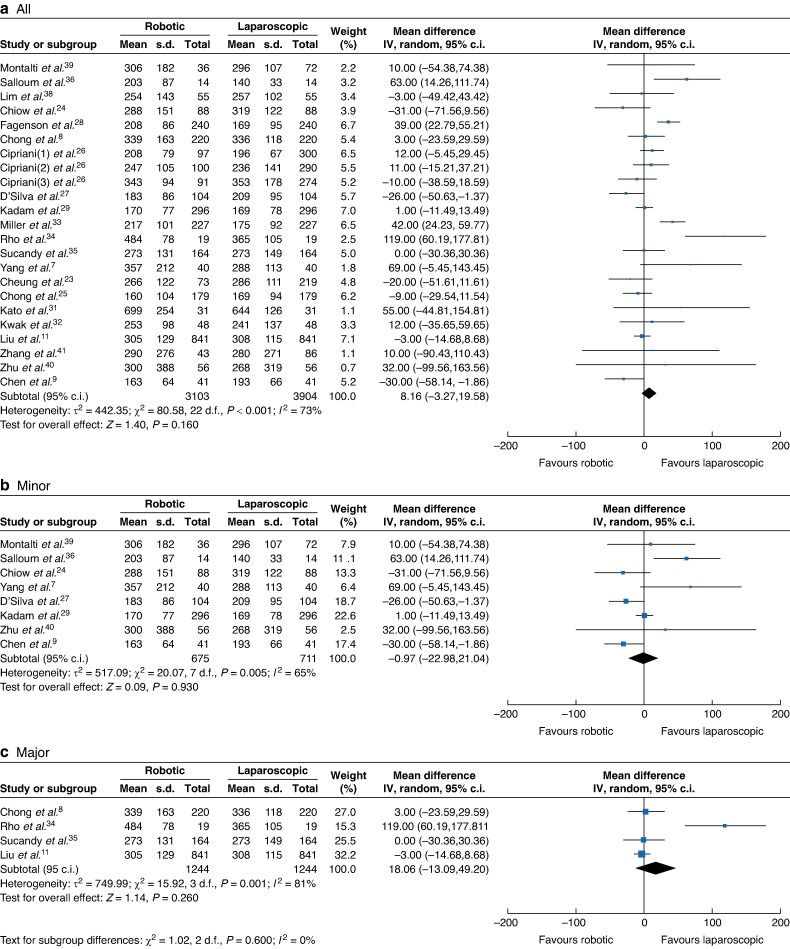
Forest plot of operative time

A total of 21 studies comprising 7007 participants^7–9,11,23–29,31–36,38–41^ (RH *versus* LH: 3103 *versus* 3904) reported the operative time. A REM was used for the meta-analysis and the results showed that there was no significant difference between the RH and LH groups (MD 8.16, 95% c.i. −3.27 to 19.58, *I*² = 73%, *P* = 0.160; *Fig. 2*).

IV, inverse variance.

**Fig. 3:**
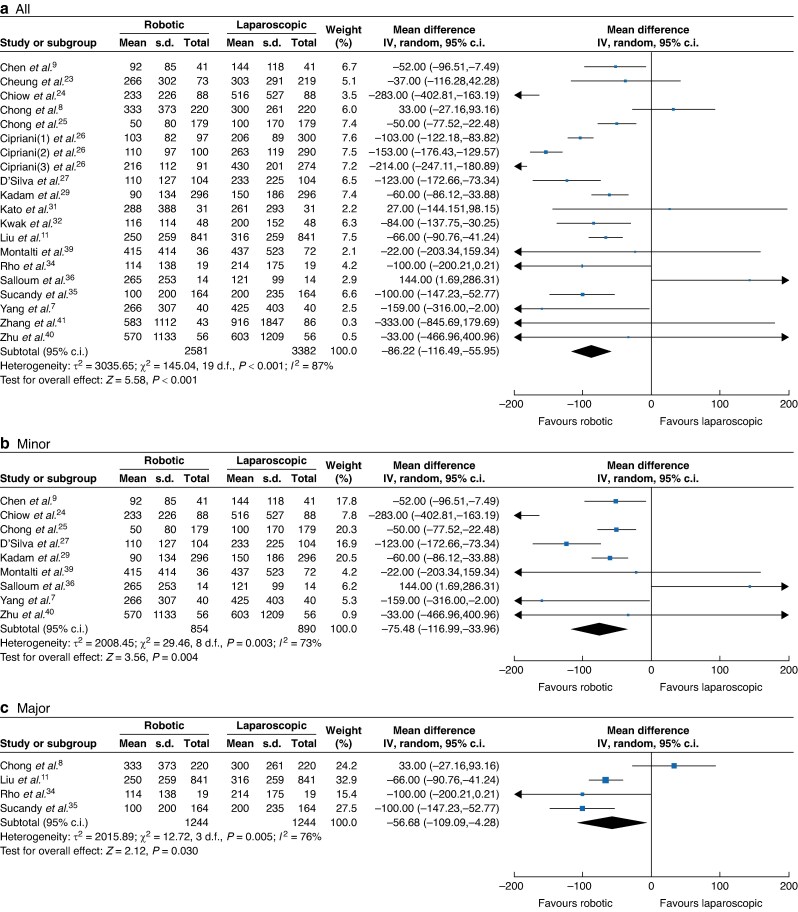
Forest plot of blood loss

**Fig. 4:**
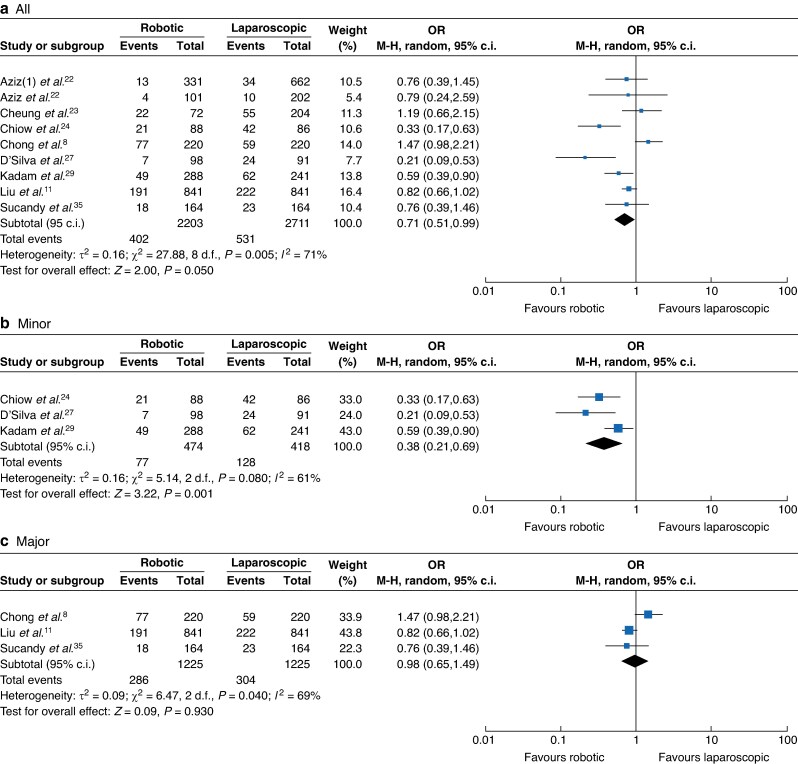
Forest plot of major blood loss

**Fig. 5:**
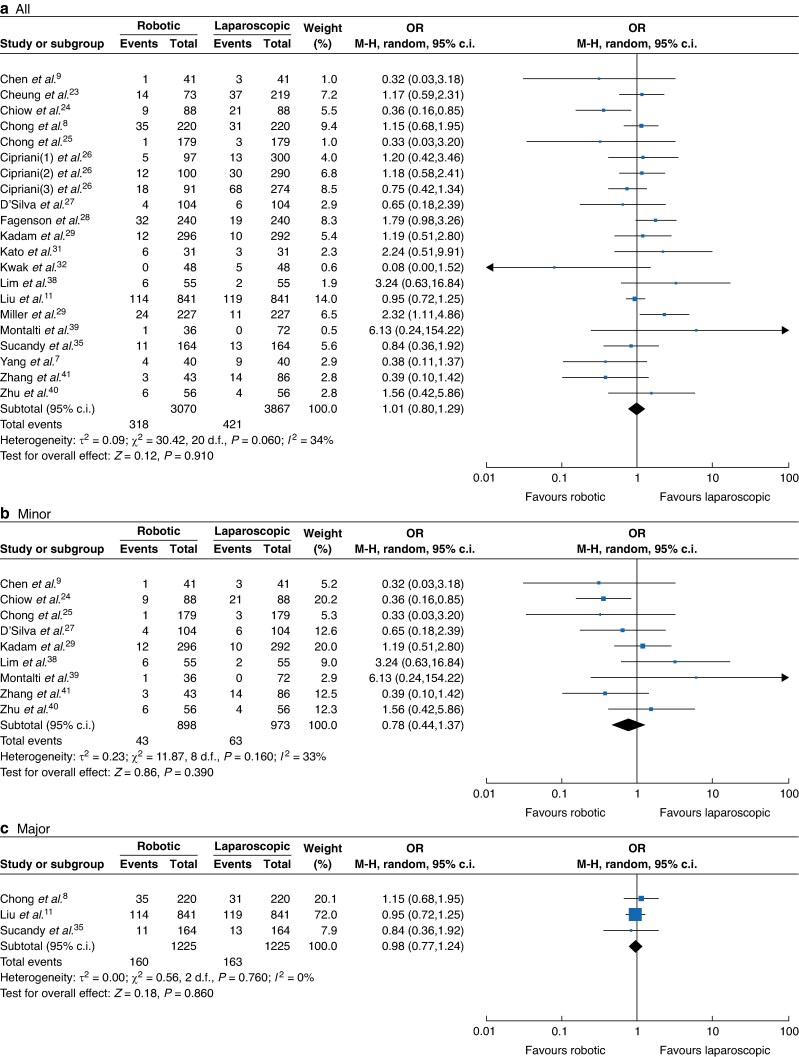
Forest plot of intraoperative blood infusion

Eighteen studies comprising 5963 participants^7–9,11,23–27,29,31,32,34–36,40–42^ (RH *versus* LH: 2581 *versus* 3382) reported blood loss. A REM was used for the meta-analysis and the results showed that blood loss of the RH group was significantly lower than that of the LH group (MD −86.22, 95% c.i. −116.49 to −55.95, *I*² = 87%, *P* < 0.001; *Fig. 3*). Similarly, nine studies comprising 4914 participants^8,11,21–24,27,29,35^ (RH *versus* LH: 2203 *versus* 2711) reported a major blood loss (>500 ml) rate and the results showed that the major blood loss (>500 ml) of the RH group was significantly lower than that of the LH group (OR 0.71, 95% c.i. 0.51 to 0.99, *I*² = 71%, *P* < 0.001; *Fig. 4*). Regarding the intraoperative blood infusion, 19 studies comprising 6937 participants^7–9,11,23–29,31–33,35,38–41^ (RH *versus* LH: 3070 *versus* 3867) showed that there was no significant difference between the RH and LH groups (OR 1.01, 95% c.i. 0.80 to 1.29, *I*² = 34%, *P* = 0.910; *Fig. 5*).

IV, inverse variance.

M-H, Mantel-Haenszel.

M-H, Mantel-Haenszel.

**Fig. 6:**
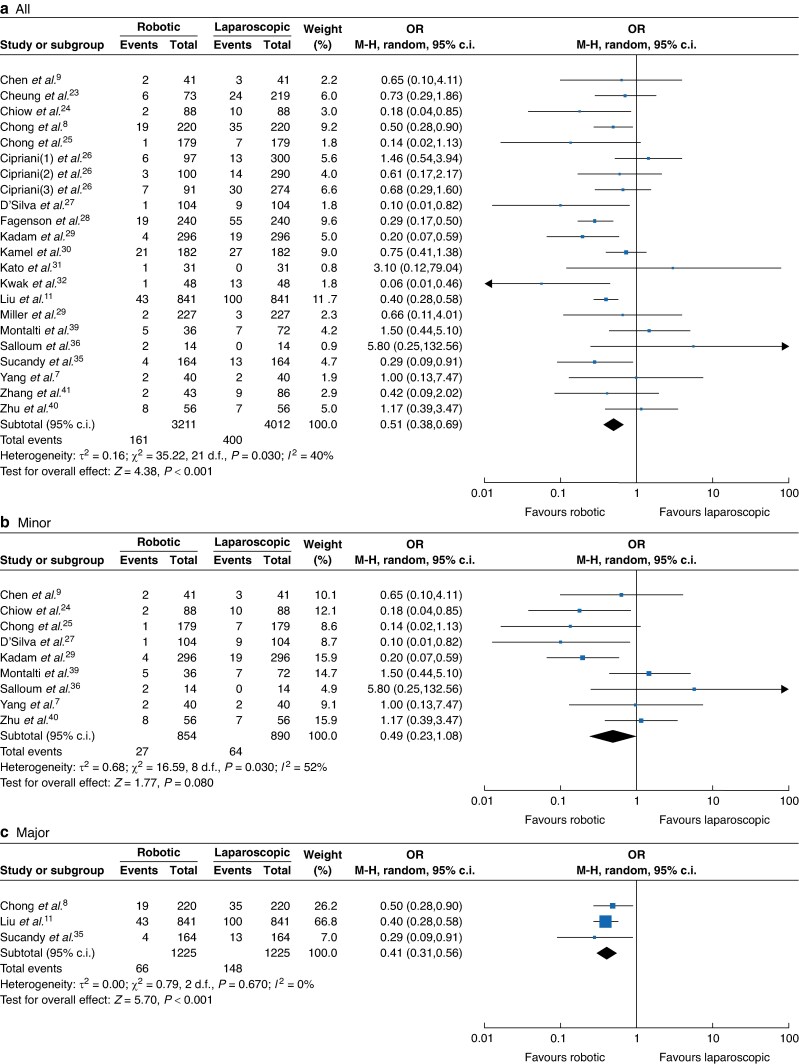
Forest plot of open conversion

Twenty studies comprising 7223 participants^7–9,11,23–33,35,36,39–41^ (RH *versus* LH: 3211 *versus* 4012) reported the open conversion rate. A REM was used for the meta-analysis and the results showed that the open conversion rate of the RH group was significantly lower than that of the LH group (OR 0.51, 95% c.i. 0.38 to 0.69, *I*² = 40%, *P* < 0.001; *Fig. 6*).

M-H, Mantel-Haenszel.

**Fig. 7:**
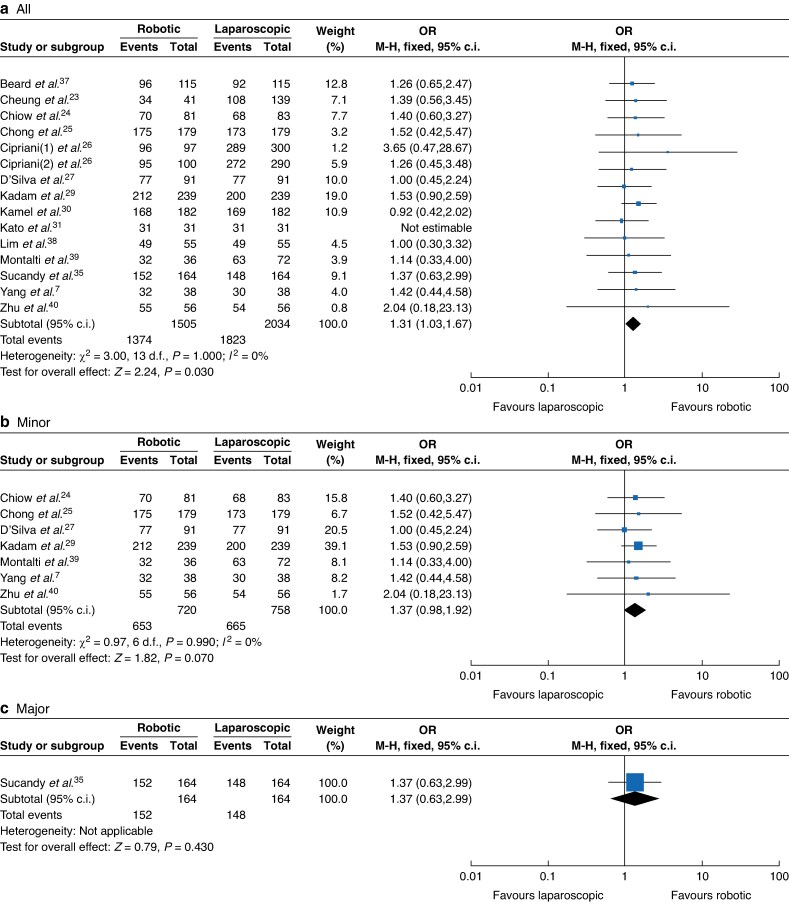
Forest plot of R0 resection

A total of 14 studies comprising 3035 participants^7,23–27,29–31,35,37–40^ (RH *versus* LH: 1505 *versus* 2034) reported the R0 resection rate. A fixed-effects model was used for the meta-analysis and the results showed that the R0 resection rate of the RH group was significantly higher than that of the LH group (OR 1.31, 95% c.i. 1.03 to 1.67, *I*² = 0%, *P* = 0.030; *Fig. 7*).

M-H, Mantel-Haenszel.

#### Postoperative outcomes

**Fig. 8:**
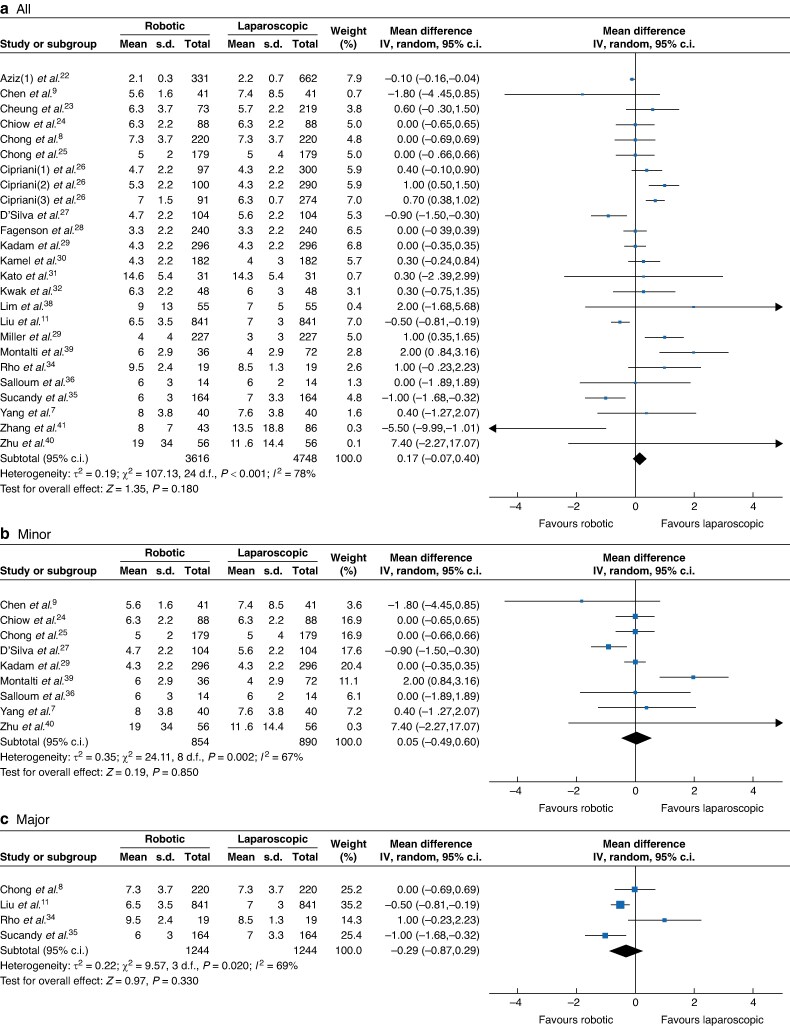
Forest plot of postoperative stay

**Fig. 9:**
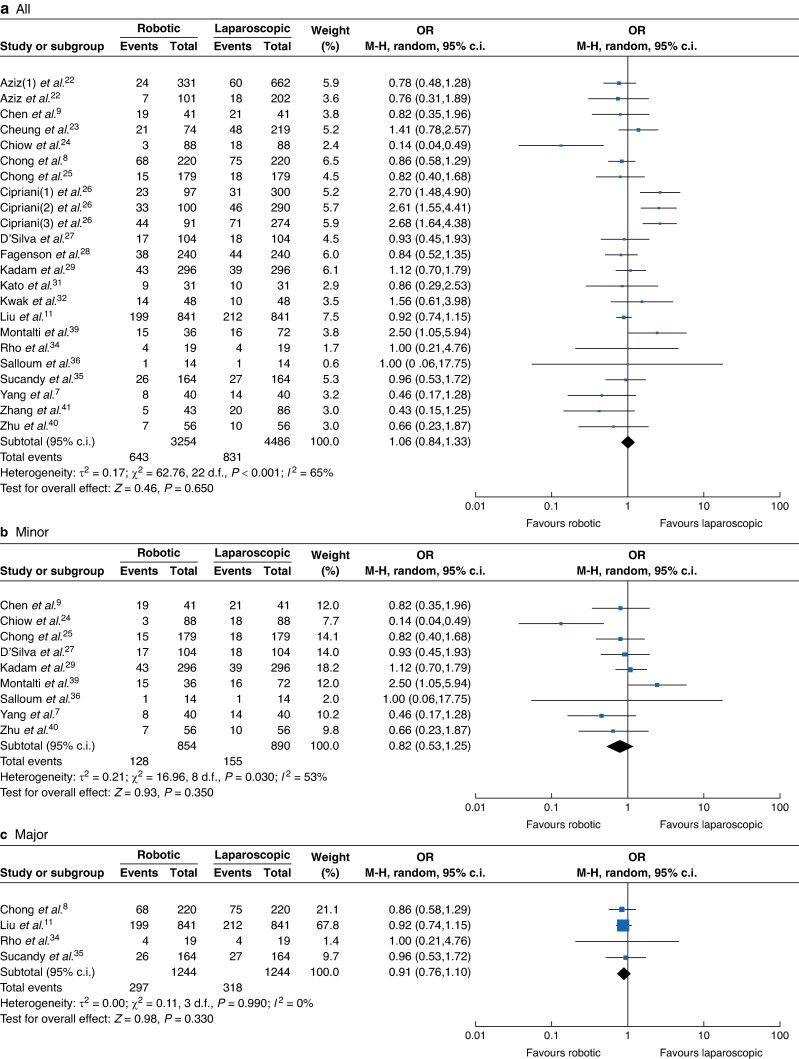
Forest plot of postoperative morbidity rate

**Fig. 10:**
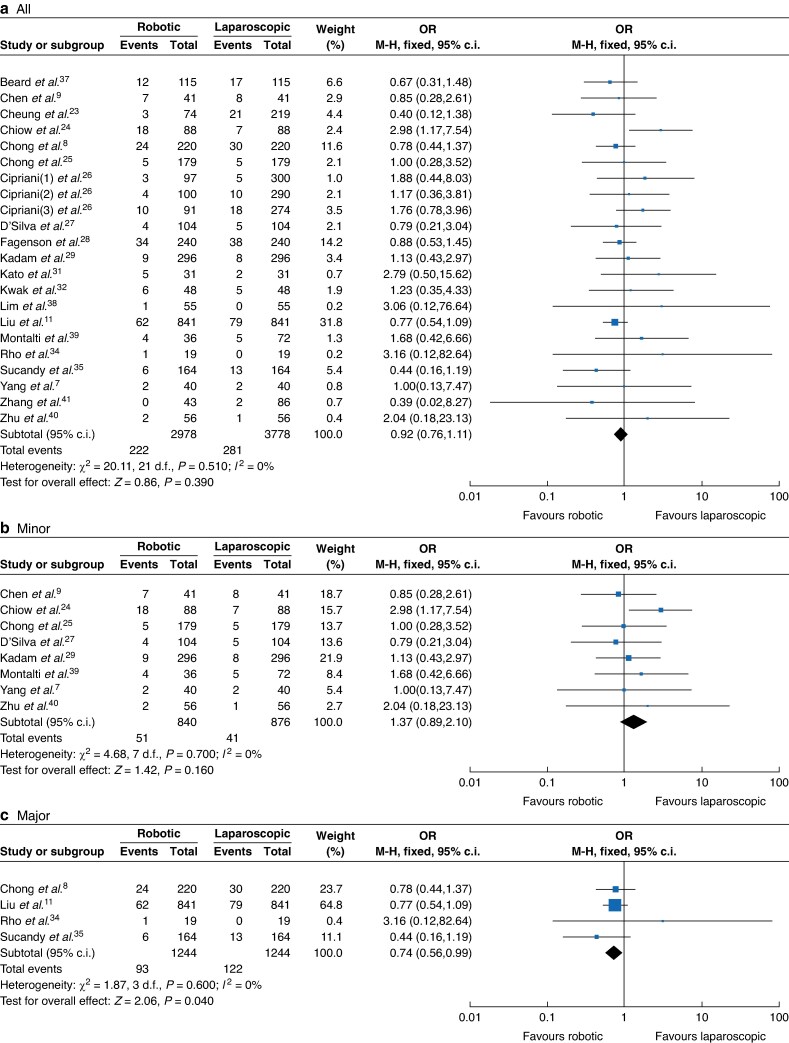
Forest plot of major morbidity rate

Concerning the postoperative outcomes, there were no significant differences between the RH and LH groups in postoperative stay (RH *versus* LH: 3616 *versus* 4748, MD 0.17, 95% c.i. −0.07 to 0.40, *I*² = 80%, *P* < 0.001; *Fig. 8*), postoperative morbidity rate (RH *versus* LH: 3254 *versus* 4486, OR 1.06, 95% c.i. 0.84, 1.33, *I*² = 65%, *P* = 0.65; *Fig. 9*) and major morbidity rate (RH *versus* LH: 2978 *versus* 3778, OR 0.92, 95% c.i. 0.76 to 1.11, *I*² = 1%, *P* = 0.440; *Fig. 10*).

IV, inverse variance.

M-H, Mantel-Haenszel.

M-H, Mantel-Haenszel.

#### Long-term outcomes

**Fig. 11:**
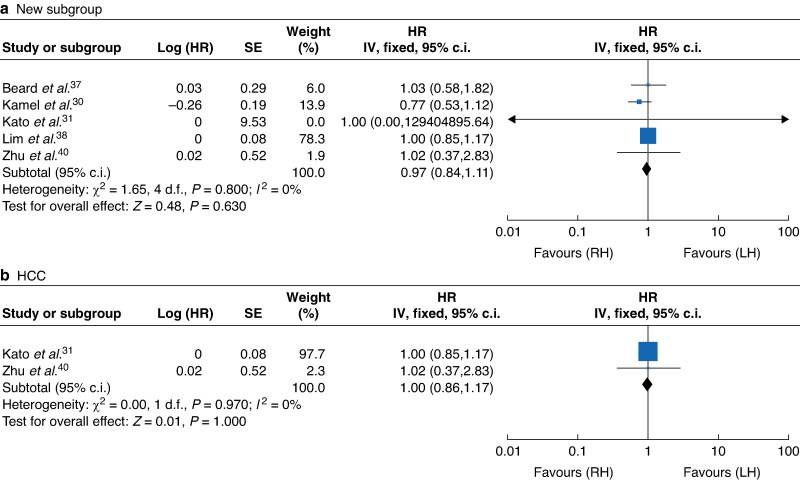
Forest plot of overall survival (OS)

HCC, hepatocellular carcinoma; IV, inverse variance; LH, laparoscopic hepatectomy; RH, robotic hepatectomy.

Five studies comprising 882 participants^30,31,37,38,40^ (RH *versus* LH: 441 *versus* 441) reported the OS. The results revealed there was no significant difference between the RH and LH groups (HR 0.97, 95% c.i. 0.84 to 1.11, *I*² = 0%, *P* = 0.630; *Fig. 11*) and there was no significant difference in hepatocellular carcinoma (HCC) subgroup (HR 1.00, 95% c.i. 0.86 to 1.17, *I*² = 0%, *P* = 1.000; *Fig. 11*).

The pooled results are summarized in *Table S2*.

#### Subgroup analysis

The pooled results of subgroup analysis including different hepatectomy type and IWATE score are summarized in *Tables S1, S4*. The pooled results showed the robotic approach had lower blood loss and a lower open conversion rate among subgroups. The robotic approach had a lower major morbidity rate in the major hepatectomy group, shorter postoperative stay in the advanced/expert level more than 80% group (as detailed in *Figs 2–10*, *Tables S3* and *S4*, and *Figs S2–8*).

#### Cost analysis

Four studies^9,22,33,36^ comprising 867 participants (RH *versus* LH: 383 *versus* 484) performed a cost-benefit analysis of RH *versus* LH. Two studies^9,36^ showed that total costs were similar between the two groups (€5436 in the RH group *versus* €4998 in the LH group; *P* = 0.51; mean(s.d.) €10 499(€1782) in the RH group *versus* €8352(€1405) in the LH group; *P* = 0.340 respectively). Miller *et al.*^33^ demonstrated that the RH group had higher total costs for each perioperative factor or postoperative complication associated than that of the LH group (€6 394 960 in the RH group *versus* €5 165 160 in the LH group; *P* < 0.010). Aziz *et al.*^22^ demonstrated the opposite (€31 427 in the RH group *versus* €34 697 in the LH group; *P* < 0.010).

## Discussion

This is one of the largest meta-analyses on this topic, with a large number of patients and specifically comparing hepatectomies using RH and LH. This present meta-analysis found that RH was associated with lower blood loss and a lower open conversion rate compared with LH. Otherwise, RH and LH had comparable safety and efficacy evidenced by the similar postoperative outcomes. In the subgroups of major and difficult hepatectomy, the lower blood loss and lower open conversion rate persisted.

Several meta-analyses have been performed to evaluate the effectiveness, safety and efficacy of RH *versus* LH^12,13,42–48^, but the analysis included a limited number of patients, or mixed hepatectomy types, or procedures with varying levels of difficulty. In addition, these studies included non-PSM retrospective studies resulting in additional confounding factors. The present meta-analysis only enrolled high-quality PSM studies and performed specific subgroup analyses, remediating the deficiencies of previous meta-analyses.

Although well-conducted RCTs were considered the ‘standard’ for treatment assessment, they were difficult and sometimes unfeasible or unethical to perform in clinical practice^49^. The use of PSM is a useful alternative to help ensure the homogeneity of the intergroup baseline data. As the PSM method reduces the impact of other confounding factors, the results should be more convincing.

Major hepatectomy is technically demanding, hindering the development of minimally invasive surgery in hepatectomy, and is associated with a significant open conversion rate^28^. Although the advantages of the robotic system might be theoretically greater in major hepatectomy, it remained unclear whether robotic or laparoscopic was better. In terms of major hepatectomy, a recent meta-analysis concluded that both techniques were equivalent when performed in selected patients and high-volume centres^14^. Another meta-analysis by Ciria *et al*. found only a slight advantage in the rate of complications and operative time for major resections with the robotic approach^13^. In our subgroup analysis, we found the robotic approach has significant advantages in terms of the estimated blood loss, open conversion rate and major morbidity rates for major hepatectomy. This may be attributed to the enlarged three-dimensional view, and the stable and flexible manoeuvrability of the robotic system, which can be of significant advantage in critical steps associated with major hepatectomy, such as hepatic vascular dissection and timely haemostasis.

The ability to stratify the difficulty of minimally invasive liver resection (including RH or LH) allows surgeons at different phases of the learning curve to tackle cases of appropriate difficulty safely. Among several difficulty scoring systems, the IWATE systems was validated in the RH and LH population^50,51^. The robotic approach was found to have a significant advantage in estimated blood loss and open conversion rate compared with that of laparoscopic. According to the IWATE scoring systems, major hepatectomy at least belonged to the advanced level and the advantage of the robotic approach for major hepatectomy was demonstrated. The increased dexterity and stability of the robotic platform proved to be more prominent for particular types of hepatectomy such as resections of posterosuperior segments.

Previous studies have suggested that the main barrier to the widespread adoption of robotic surgery is limited access to the technology due to its high cost^52,53^. A recent review concluded that the cost per robotic procedure is approximately 20–25% higher than laparoscopic resections^13^. In four studies of this present review^9,22,33,36^, a cost-benefit analysis was performed with rather different conclusions. Although robotic surgery needs the additional fee of the robotic system, there was a financial benefit when considering the subsequent costs related to therapy for morbidity rate, postoperative stay and readmission compared with that of laparoscopic^22^. With new models of surgical robots coming on to the market, the cost of the robotic platform is likely to decrease^8^. Nonetheless, given the current significant advantages demonstrated by RH, it would be recommended to perform RH in patients with a high morbidity rate risk.

This study had several limitations. The retrospective nature of the included studies was inevitably associated with information and selection bias. Although PSM analysis was performed to mitigate this, residual bias is likely to remain and certain data, such as experience level, were not available and were unable to be matched. A high degree of heterogeneity was found among the studies. Despite the use of subgroup analysis, significant heterogeneity remained, perhaps due to the complexity of the population compositions and centre differences. Most of the included studies were international multicentre investigations and although this increased the generalizability of the results, there was still considerable heterogeneity in terms of patient selection, surgeon/centre experience, surgical technique and perioperative management. Caution must be used in interpreting some outcomes which were analysed by few studies (for example cost analysis). Some results could be distorted by the fact that robotic surgical techniques are usually performed by experienced surgeons.

This study found that some surgical outcomes were similar between RH and LH, but RH had advantages in terms of lower blood loss and conversion to open, as well as in difficult hepatectomies. More studies need to clarify the comparative benefits of RH and LH in particular patients in the future, such as in patients with posterior segment resection or cirrhosis.

## Supplementary Material

zrae141_Supplementary_Data

## Data Availability

All data generated or analysed during this study are included in this article (and its *Supplementary material*).
