# Supplementary material for: Robotic versus laparoscopic hepatectomy: meta-analysis of propensity-score matched studies
Source: BJS Open. 2025 Apr 1;9(2):zrae141. doi: 10.1093/bjsopen/zrae141 (PMC11957917; doi:10.1093/bjsopen/zrae141)

**Robotic Versus Laparoscopic Hepatectomy: Meta-Analysis of Propensity-Score Matched Studies**

Piao Wang^#,1^, Dan Zhang^#,2^, Bin Huang^1^, Wen-hao Zhou^1^, Chang-song Wang^1^, Shao-yong Zhao^1^, Song Su^*,3^, Xiao-zhong Jiang^*, 1^

1 Department of Hepatobiliary and Pancreatic Surgery, The Second People's Hospital of Yibin, Sichuan, China. 644002.

2 Department of Thyroid and Breast Surgery, The Third People's Hospital, Yibin, Sichuan, China. 644002.

3 Department of General Surgery (Hepatobiliary Surgery), The Affiliated Hospital of Southwest Medical University, Luzhou, 646000, China

* Correspondence: Song Su, [13882778554@163.com](mailto:jiangxiaozhong66@163.com); Xiao-zhong Jiang, [jiangxiaozhong66@163.com](mailto:jiangxiaozhong66@163.com).

**Supplementary Materials - Index**

**Supplementary Figure**

**Fig S1** a funnel plot of open conversion; **Fig S1b** funnel plot of postoperative day. Pag 2

**Supplementary Table**

**Table S1 The Quality of Retrospective Cohort Studies** Pag 3

**Table S2 Pooled Results of all patients** Pag 4

**Table S3 Pooled Results of subgroup of different hepatectomy type** Pag 5

**Table S4 Pooled Results of subgroup of** **different Iwate score** Pag 6

**Fig S2-8 The forest plot of subgroup analysis (advanced/expert level >80%)**

**Fig S2 The forest plot of operative time** Pag 7

**Fig S3 The forest plot of blood loss** Pag 8

**Fig S4 The forest plot of blood loss(≥500ml)** Pag 9

**Fig S5 The forest plot of intraoperative blood infusion** Pag 10

**Fig S6 The forest plot of open conversion** Pag 11

**Fig S7 The forest plot of R0 resection** Pag 12

**Fig S8 The forest plot of postoperative stay** Pag 13

**Fig S9 The forest plot of postoperative morbidity** Pag 14

**Fig S10 The forest plot of major morbidity** Pag 15

**The PRISMA checklist** Pag 16-17

**
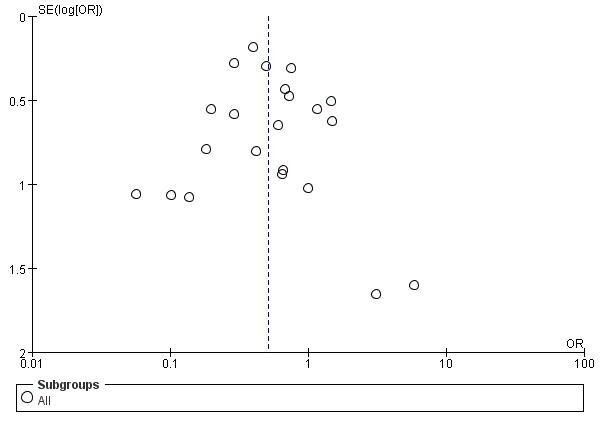
**

**Fig S1 a,** funnel plot of open conversion;


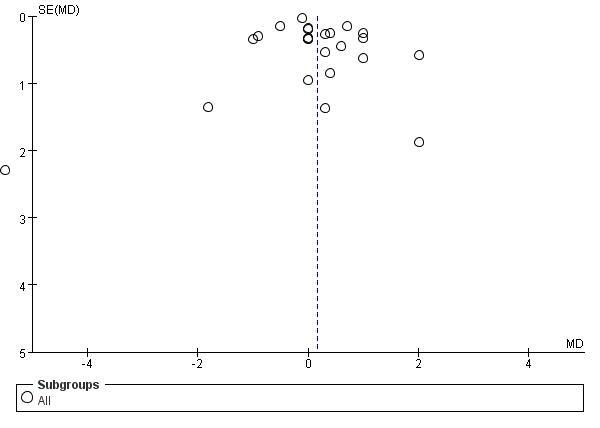


**Fig S1b,** funnel plot of postoperative day

| **Studies** | **Selection** | **Comparability** | **Exposure** | **Scores** |
| --- | --- | --- | --- | --- |
| Montalti 2016 | ★★ | ★★ | ★★★ | 7 |
| Salloum 2016 | ★★ | ★★ | ★★★ | 7 |
| Lim 2019 | ★★ | ★★ | ★★★ | 7 |
| Beard 2020 | ★★ | ★★ | ★★★ | 7 |
| Chiow 2021 | ★★ | ★★ | ★★★ | 7 |
| Fagenson 2021 | ★★★ | ★★ | ★★★ | 8 |
| Aziz 2022 | ★★★ | ★★ | ★★★ | 8 |
| Aziz 2022（2） | ★★★ | ★★ | ★★★ | 8 |
| Chong 2022 | ★★ | ★★ | ★★★ | 7 |
| Cipriani 2022 | ★★★ | ★★ | ★★★ | 8 |
| D'Silva 2022 | ★★ | ★★ | ★★★ | 7 |
| Duong 2022 | ★★★ | ★★ | ★★★ | 8 |
| Kadam 2022 | ★★ | ★★ | ★★★ | 7 |
| Kamel 2022 | ★★★ | ★★ | ★★★ | 8 |
| Miller 2022 | ★★★ | ★★ | ★★★ | 8 |
| Rho 2022 | ★★★ | ★★ | ★★★ | 8 |
| Sucandy 2022 | ★★ | ★★ | ★★★ | 7 |
| Yang 2022 | ★★ | ★★ | ★★★ | 7 |
| Chen 2023 | ★★★ | ★★ | ★★★ | 8 |
| Cheung 2023 | ★★ | ★★ | ★★★ | 7 |
| Chong 2023 | ★★ | ★★ | ★★★ | 7 |
| Kato 2023 | ★★★ | ★★ | ★★★ | 8 |
| Kwak 2023 | ★★ | ★★ | ★★★ | 7 |
| Liu 2023 | ★★ | ★★ | ★★★ | 7 |
| Zhang 2023 | ★★★ | ★★ | ★★★ | 8 |
| Zhu 2023 | ★★★ | ★★ | ★★★ | 8 |

**Table S1 The Quality of Retrospective Cohort Studies**

**Table S2 Pooled Results of all patients**

**Table S3 Pooled Results of subgroup of different hepatectomy type**

**Table S4 Pooled Results of subgroup of** **different Iwate score**


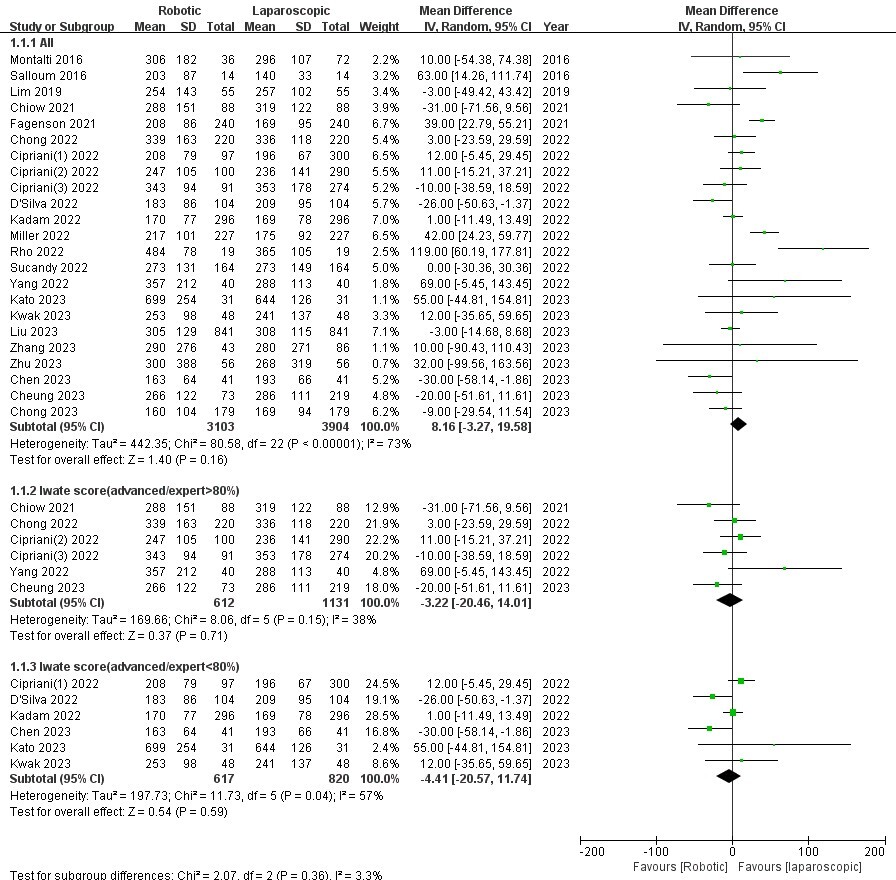


**Fig S2 The forest plot of operative time**


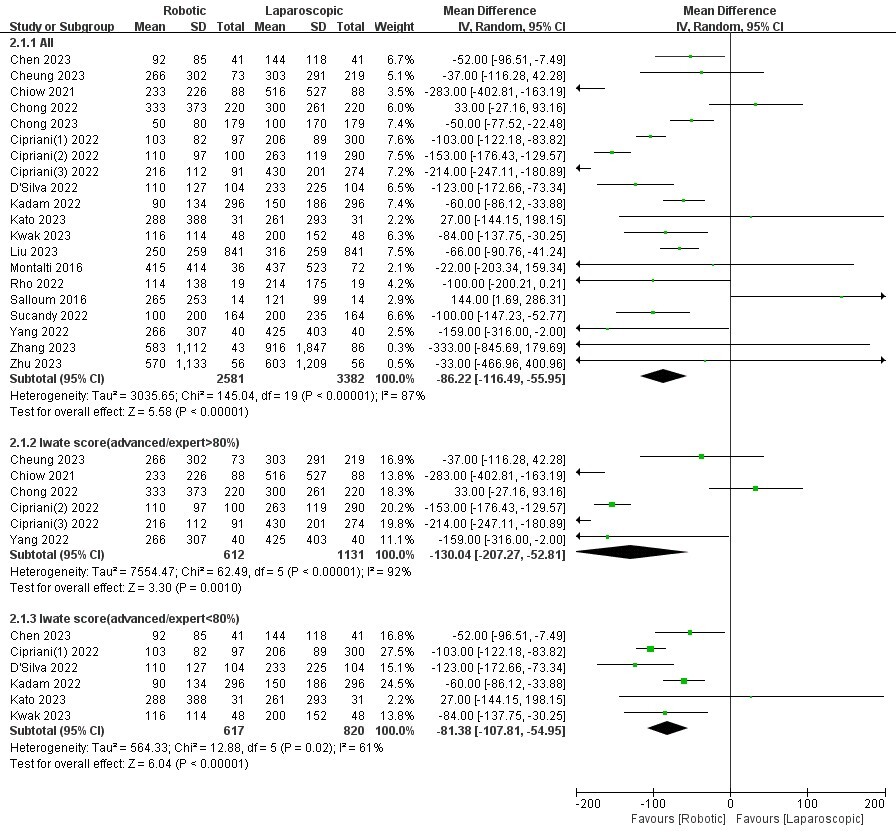


**Fig S3 The forest plot of blood loss**


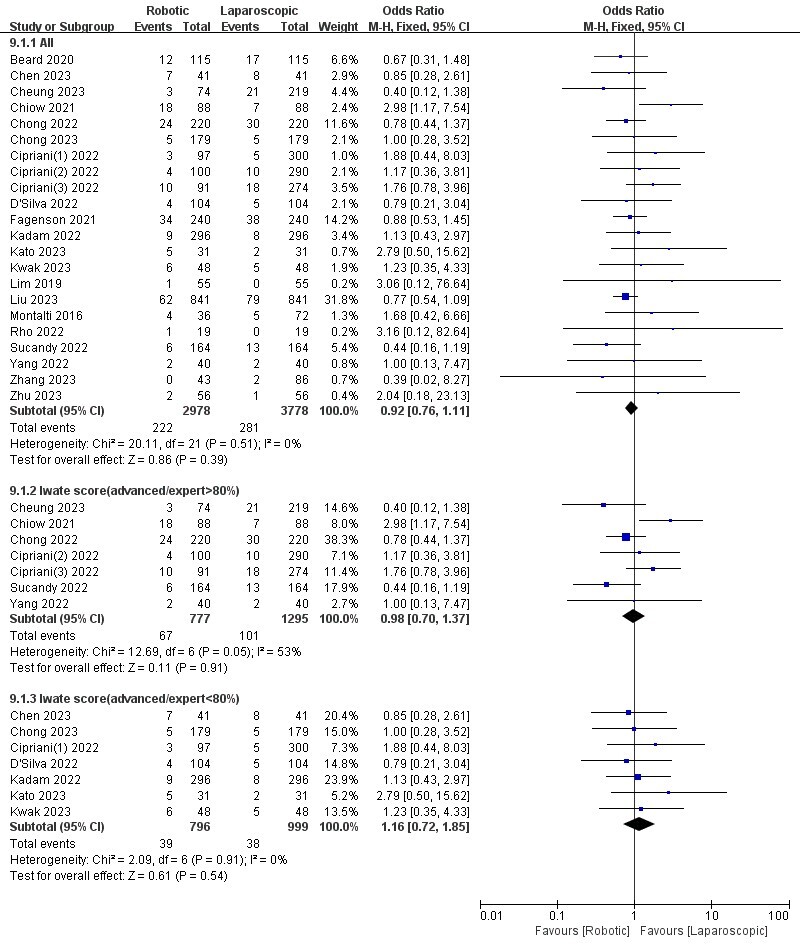


**Fig S4 The forest plot of major blood loss(≥500ml)**


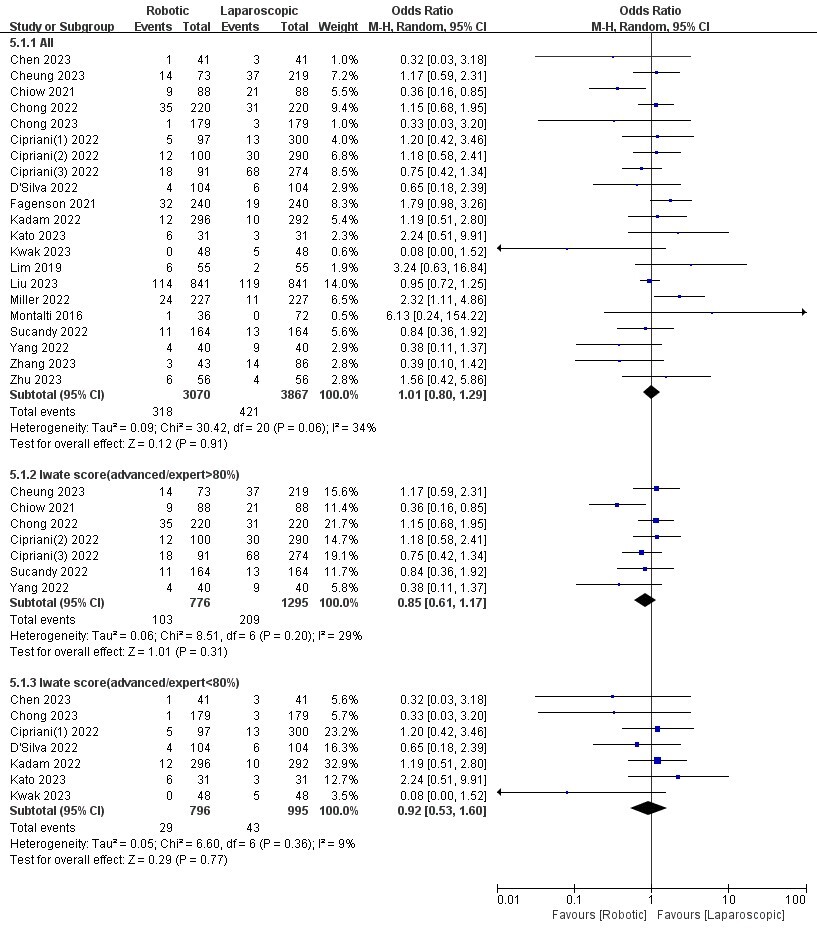


**Fig S5 The forest plot of intraoperative blood infusion**


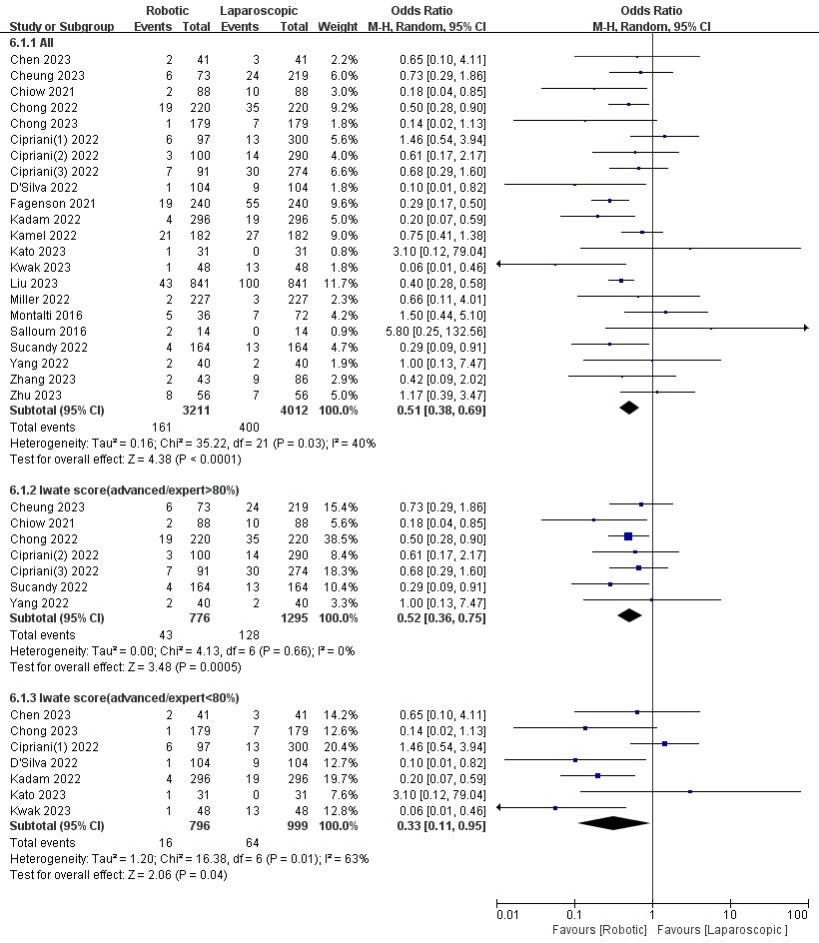


**Fig S6 The forest plot of open conversion**


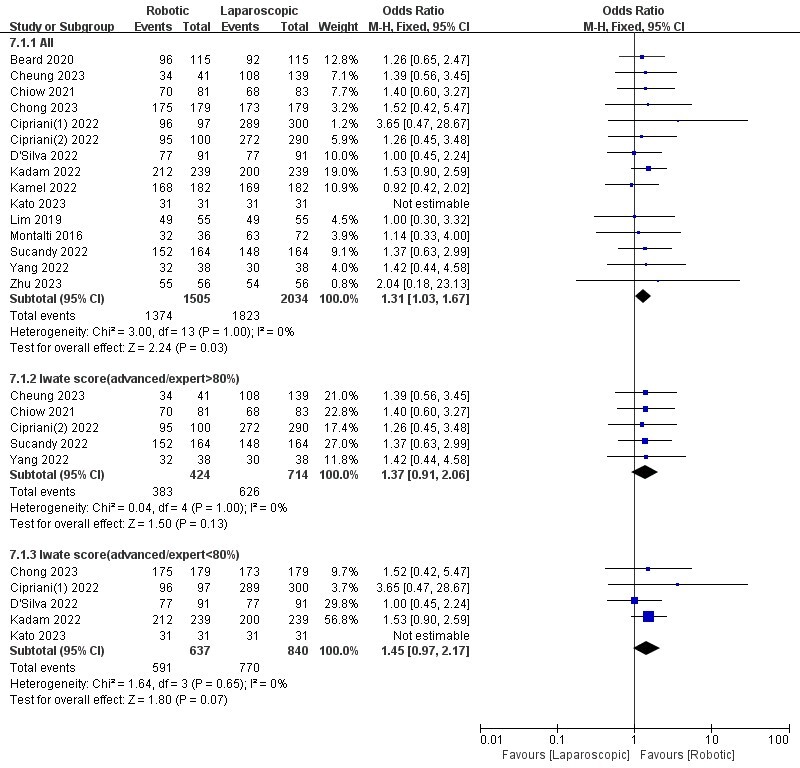


**Fig S7 The forest plot of R0 resection**


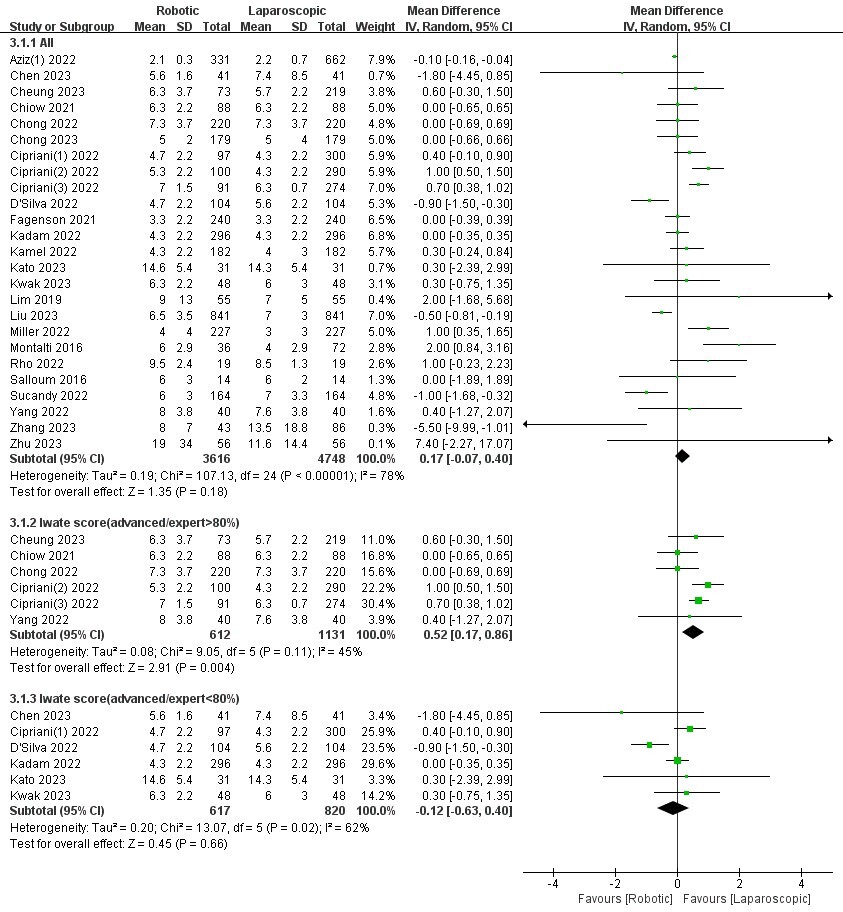


**Fig S8 The forest plot of postoperative stay**


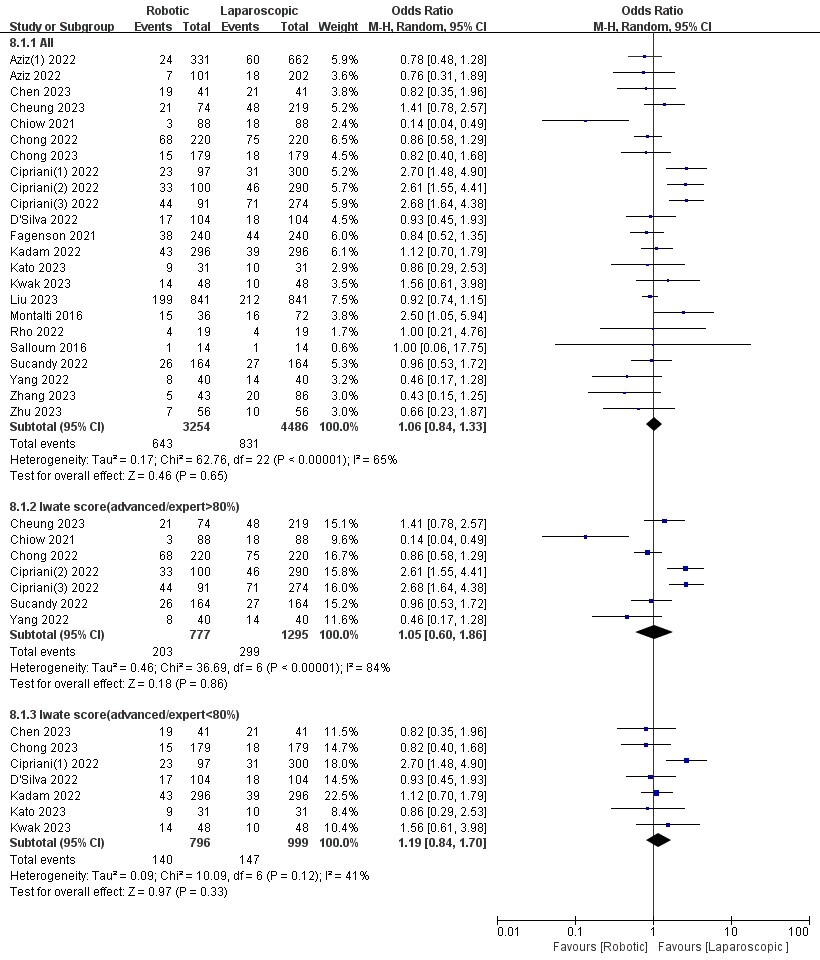


**Fig S9 The forest plot of postoperative morbidity**


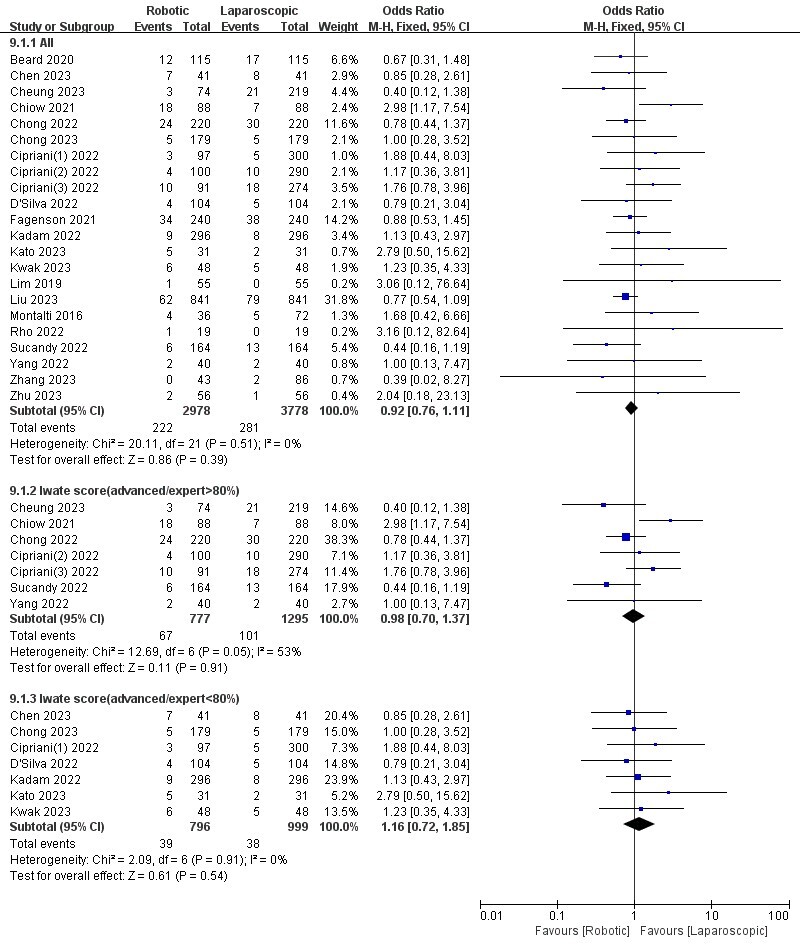


**Fig S10 The forest plot of major morbidity**


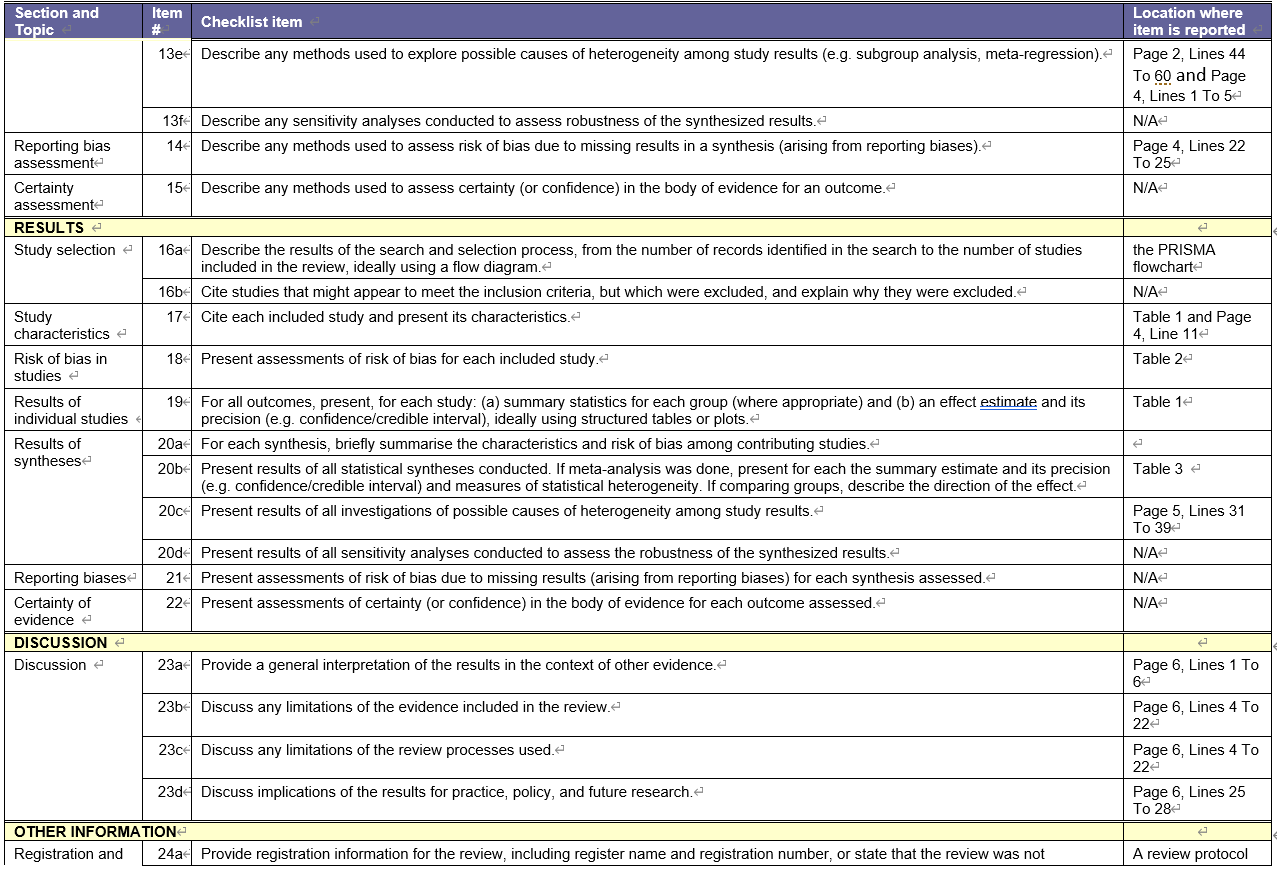

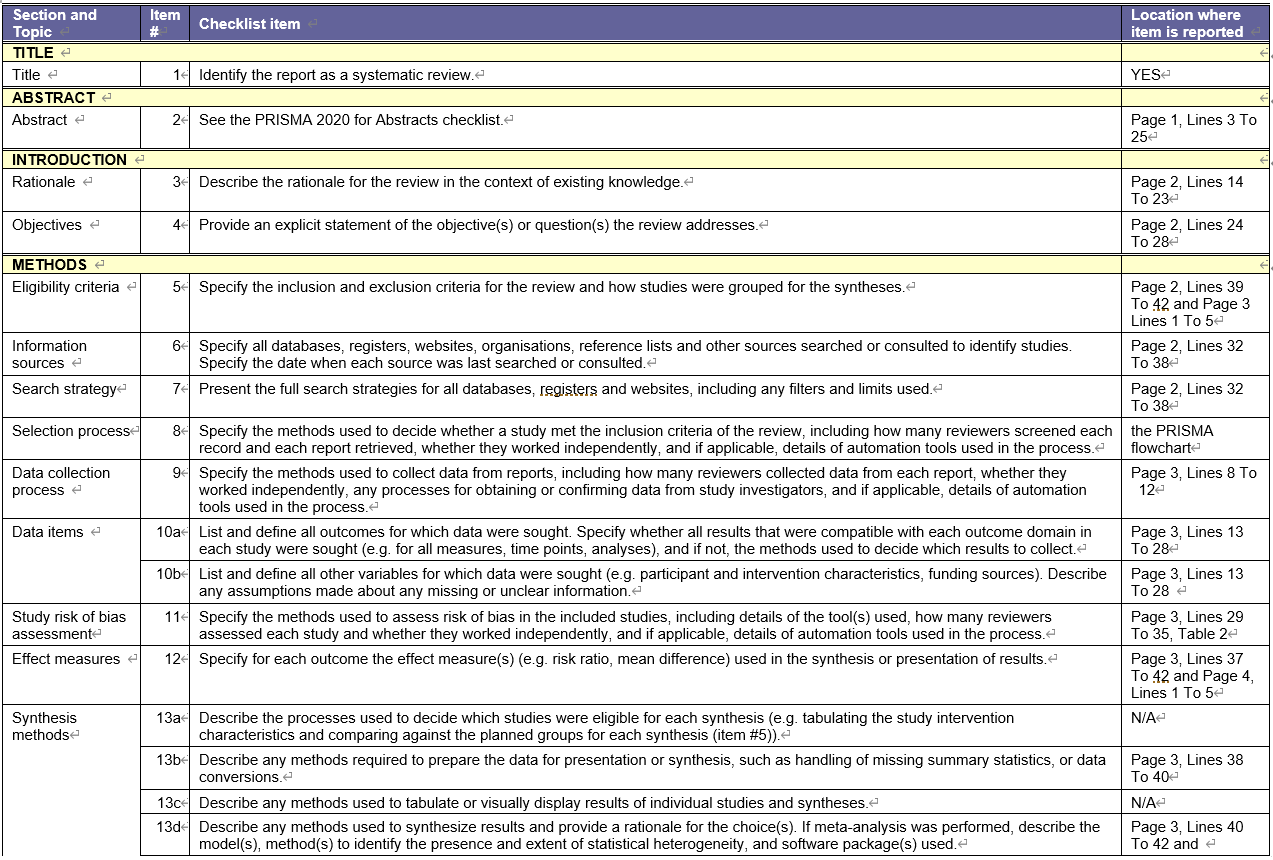


**The PRISMA checklist**
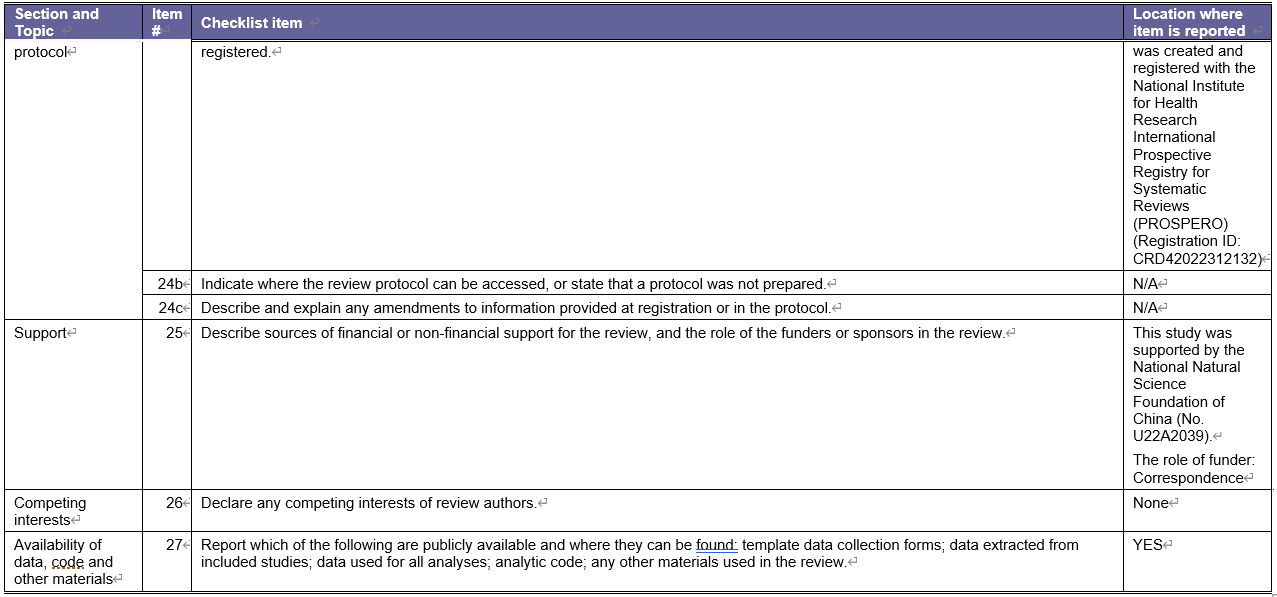

Supplement: zrae141_Supplementary_Data [file zrae141_supplementary_data.docx]
